# COVID-19 onslaught is masking the 2021 dengue outbreak in Dhaka, Bangladesh

**DOI:** 10.1371/journal.pntd.0010130

**Published:** 2022-01-20

**Authors:** Mohammad Sorowar Hossain, Robed Amin, Abdullah Al Mosabbir

**Affiliations:** 1 Department of Emerging and Neglected Diseases, Biomedical Research Foundation, Dhaka, Bangladesh; 2 School of Environment and Life Sciences, Independent University, Bangladesh, Dhaka, Bangladesh; 3 Non-communicable Disease Control, Directorate General of Health Services (DGHS), Dhaka, Bangladesh; Jouf University, Kingdom of Saudi Arabia, SAUDI ARABIA

## Background

Bangladesh, a South Asian country with over 165 million people, is currently confronting the worst phase of the Coronavirus Disease 2019 (COVID-19) pandemic since the official declaration on 8 March 2020, with a record surge in cases and deaths. After ravaging the neighboring countries (India and Nepal), the highly contagious delta variant has been spreading rapidly throughout Bangladesh. While more than 110,000 confirmed cases and 17,894 deaths had been reported in Bangladesh as of 19 July 2021, the ongoing third wave (May to 1 July 2021) has already affected over 300,000 and claimed more than 6,000 lives [1]. However, this is merely an underrepresentation of the actual scenario due to a low level of testing in Bangladesh [2]. To contain the infection, a nationwide lockdown (limited office openings and transport services) has been imposed since 5 April 2021. Later, the government has deployed the military to implement the strictest version of lockdown (all offices and transports were closed) from 1 July 2021. However, the onslaught of this wave is yet to show any sign of slowing down. It is important to mention here that unlike in developed countries, lockdowns were not practically implemented in Bangladesh because of poor general health awareness, public reluctance, and other socioeconomic factors. As a result, almost all hospital beds dedicated to severe and critical patient care in the country, especially those in Dhaka, are currently occupied by patients of COVID-19 [3]. On the other hand, the country’s vaccine drive is still at an early stage; only around 3% (as of 19 July 2021) of the country’s population has received 2 doses. Now vaccination coverage has reached to 20.32% as of 24 November 2021 [4].

Bangladesh is also a hyperendemic country for dengue infection [5]. In recent years, Bangladesh witnessed an upsurge of vector-borne disease outbreaks in Dhaka city, especially dengue and chikungunya. The first-ever massive outbreak of chikungunya in 2017 was followed by the largest outbreak of dengue in 2019 [6,7]. While dengue was causing small outbreaks in every few years since its first appearance in 2000, the 2019 outbreak broke all records and caused the hospitalization of more than 100,000 dengue patients, affecting every age group and socioeconomic stratum [7]. The healthcare system almost collapsed, causing widespread panic in the city. Unfortunately, dengue outbreaks are expected in Dhaka city during every monsoon season because of the high population density, unplanned urbanization, and favorable climatic conditions for vectors [8,9]. Given the current context of the COVID-19 pandemic, a concurrent dengue outbreak is expected to bring catastrophe. This concurrent outbreak in a resource-constraint country is likely to be a unique event. Therefore, experiences from these simultaneous outbreaks would be vital for managing future infectious disease outbreaks elsewhere in the world.

## Has the dengue outbreak started in Dhaka city?

We have collected data of hospitalized dengue cases from the Directorate General of Health Services (DGHS), Bangladesh, to investigate the potential dengue outbreak in 2021 [10]. Month-wise cumulative data over a period of 2010 to 2020 showed that nearly 80% of all dengue cases were reported in the months of July to September (monsoon) with a peak in August (S1 Fig and S1 Table). A similar trend of a typical dengue outbreak can be observed this year based on available data for Dhaka city (January to 15 July 2021). Until the end of May, only a few hospitalized cases (*n =* 103) were reported. However, the number of dengue cases doubled in June (*n =* 272) and continued to rise (*n* = 767) in a similar trend up to 17 July 2021, and 2 unofficial death cases were reported. While DGHS reported 1,193 dengue cases amid the COVID-19 pandemic last year (2020), the trend was not similar to previous large outbreaks, although a late spike was reported in November 2020.

The true magnitude of dengue infection is possibly concealed due to severely disrupted healthcare systems to tackle the ongoing COVID-19 crisis. In addition, misdiagnosis or delay in diagnosis of dengue is conceivable because of the similarities in clinical manifestations of these 2 diseases [11]. Naturally, all febrile cases are being suspected and treated as COVID-19 infection until proved otherwise. Therefore, it is conceivable that majority dengue cases with mild to moderate symptoms could have escaped detection due to current COVID-19 surge. Besides, the dengue surveillance system is not well established since a functional referral system and systematic record-keeping of medical history are not well practiced in Bangladesh. For instance, where the World Health Organization (WHO) estimated more than 300,000 dengue patients in July 2019, only 7,179 cases were reported officially [12]. Most importantly, DGHS collects data only from 41 healthcare centers out of several hundred private hospitals/clinics in Dhaka for dengue surveillance. Notably, the existing surveillance mainly concentrates on Dhaka, while studies suggest that dengue has spread to some nonendemic districts of Bangladesh during the 2019 outbreaks [13,14]. Despite Dhaka megacity being hyperendemic for dengue, an integrated vector control policy has not been established yet. The city corporations lack adequate infrastructure and manpower [15,16]. Besides, community engagement is also lacking. Moreover, lockdowns and/or restrictions due to the 18-month long COVID-19 pandemic has further exacerbated the capabilities of vector control efforts [17]. Consequently, this year (2021), more *Aedes* mosquitoes are hatched, leading to a dengue outbreak of much higher magnitude than that of 2020. People staying home due to lockdown may become easy prey for mosquitoes. Interestingly, movement restrictions (lockdowns) resulted higher incidence of dengue cases in Singapore, while the opposite impact was reported in Sri Lanka [18,19].

## Why are concurrent outbreaks of 2 viruses a matter of serious concern in a crowded city like Dhaka?

Dengue is the most dangerous arboviral disease in the world due to its associated high morbidity and mortality [20]. Recently, it has reemerged as one of the major global public health concerns with a dramatic increase in the frequency of outbreaks and severity of clinical manifestations. Unfortunately, there is no specific treatment for dengue infection. Severely affected patients require proper supportive care and more critical patients require intensive care unit (ICU) support [21]. Likewise, patients with critical COVID-19 also require ICU support. Therefore, simultaneous outbreaks of dengue and COVID-19 can potentially worsen the outcome of both infections by limiting the availability of emergency services [11]. Besides, coinfection with dengue and COVID-19 is now commonly reported in the literature and is associated with high mortality [22].

Dhaka is the most crowded megacity (over 47,000 inhabitants per square kilometer) in the world, with nearly 20 million population, where resources are minimal compared to developed countries [23]. This city is the cornerstone of critical medical care services in Bangladesh as it holds most of the country’s tertiary care settings and ICU facilities. For instance, out of the total 1,217 ICU beds available in the country, 839 are in Dhaka [24]. The ongoing surge of COVID-19 pandemic is putting massive strain to the emergency services of all the major hospitals in Dhaka. Therefore, a full-scale dengue outbreak would bring a debacle to the already overwhelmed healthcare system of the city, leading to a massive increase in the morbidity and mortality of both infections.

## Current dengue situation in Bangladesh

**Fig 1:**
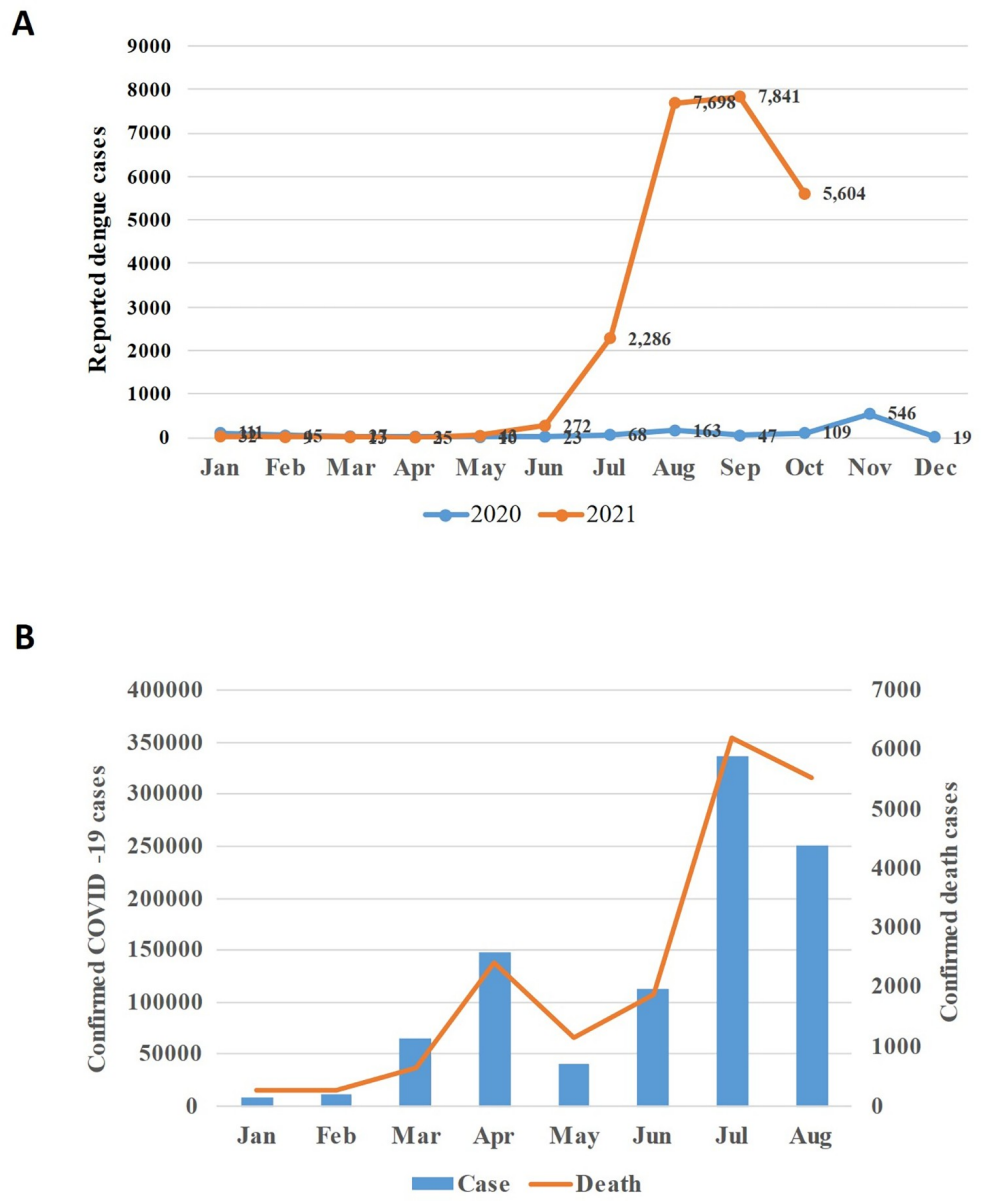
Trend of dengue and COVID-19 case reporting. **(A)** Number month-wise hospitalized dengue cases 2020 and 2021. **(B)** Number of confirmed COVID-19 cases by months in 2021. COVID-19, Coronavirus Disease 2019.

While we prepared this manuscript, the dengue outbreak had just started, and we predicted an eminent large-scale outbreak. As of 17 November 2021, more data on dengue outbreak and COVID-19 pandemic became available. These data confirm our prediction. The deadly delta variant of Severe Acute Respiratory Syndrome Coronavirus 2 (SARS-CoV-2) surge peaked at the end of July and first week of August 2021 and then started to decline gradually (Fig 1B). Currently, COVID-19 outbreak is under control with a detection rate of about 6% [25]. Consequently, the dengue outbreak became unmasked. The number of hospitalized dengue cases increased over 3-fold in August (*n =* 7,689) as compared to July (*n* = 2,286) (Fig 1A). As of 17 November, 26,000 dengue cases were reported with 98 deaths [26]. So far, the case fatality rate seems higher in this outbreak (98/26,000) as compared to the 2019 massive outbreak (179/102,354) (S1 Table). This is alarming and warrants further research. Besides, the infection is still showing an upward trend. The average daily cases are higher in September than in August (294 versus 248) [26].

## Recommendations

It is obvious that public awareness about the deadly dengue and COVID-19 outbreaks is very low in Bangladesh. Therefore, raising community awareness must be of utmost priority. Despite Bangladesh being a dengue-endemic country (outbreaks are expected every year), control programs are mostly focused on emergency responses rather than prevention. It is therefore necessary to emphasize year-round vector control and surveillance. Dengue outbreaks are getting more frequent in recent years in Bangladesh. This is very alarming for a densely populated country like Bangladesh. Hence, strengthening healthcare capacity by allocation of resources are needed to ensure continuous surveillance and early recognition of coepidemics like this. Besides, revision of both dengue and COVID-19 guidelines is necessary to aid healthcare workers in dealing with such challenging situations. As dengue could spread to nonendemic regions of the country, local health authorities should be alerted and prepared accordingly at district levels. From a long-term perspective, efforts to identify the ecological niche of the *Aedes* mosquito should be emphasized. For a densely populated country, information-based decision-making (such as robust surveillance system and molecular tracking of genotypes of dengue virus) and integrated vector control strategies could be more effective than the random efforts of the city corporations in the major cities. Emerging infectious diseases usually strike in a limited geographical area first, although they can quickly become a global issue in the interconnected world. Resource-limited countries like Bangladesh are not capable of fighting and containing high magnitude infectious disease outbreaks alone. Therefore, international cooperation and support are necessary to prevent the spread of emerging and reemerging disease outbreaks around the world.

## Supporting information

S1 Table Number of reported hospitalized dengue cases by months between 2010 and 2020 and yearly deaths. (DOCX)

S1 Fig Cumulative trend of dengue cases over a period of 2010–2020. (DOCX)
