# Supplementary material for: COVID-19 onslaught is masking the 2021 dengue outbreak in Dhaka, Bangladesh
Source: PLoS Negl Trop Dis. 2022 Jan 20;16(1):e0010130. doi: 10.1371/journal.pntd.0010130 (PMC8775334; doi:10.1371/journal.pntd.0010130)
Supplement: S1 Table — (DOCX) [file pntd.0010130.s001.docx]

**S1 Table**: Number of reported hospitalized dengue cases by months between 2010 and 2020 and yearly deaths.

| **Months** | **2010** | **2011** | **2012** | **2013** | **2014** | **2015** | **2016** | **2017** | **2018** | **2019** | **2020** | **2021** |
| --- | --- | --- | --- | --- | --- | --- | --- | --- | --- | --- | --- | --- |
| **January** | 0 | 0 | 0 | 6 | 15 | 0 | 13 | 92 | 26 | 38 | 111 | 32 |
| **February** | 0 | 0 | 0 | 7 | 7 | 0 | 3 | 58 | 7 | 18 | 45 | 9 |
| **March** | 0 | 0 | 0 | 3 | 2 | 2 | 17 | 36 | 19 | 17 | 27 | 13 |
| **April** | 0 | 0 | 0 | 3 | 0 | 6 | 38 | 73 | 29 | 58 | 25 | 3 |
| **May** | 0 | 0 | 0 | 12 | 8 | 10 | 70 | 134 | 52 | 193 | 10 | 43 |
| **June** | 0 | 61 | 10 | 50 | 9 | 28 | 254 | 267 | 295 | 1884 | 23 | 272 |
| **July** | 61 | 255 | 129 | 172 | 82 | 171 | 926 | 286 | 946 | 16253 | 68 | 2286 |
| **August** | 183 | 691 | 122 | 339 | 80 | 765 | 1451 | 346 | 1796 | 53636 | 163 | 7698 |
| **September** | 120 | 193 | 246 | 385 | 76 | 965 | 1544 | 430 | 3087 | 16856 | 47 | 7841 |
| **October** | 45 | 114 | 107 | 501 | 63 | 869 | 1077 | 512 | 2406 | 8143 | 109 | 5604 |
| **November** | 0 | 36 | 27 | 218 | 22 | 271 | 522 | 409 | 1192 | 4011 | 546 |  |
| **December** | 0 | 9 | 0 | 53 | 11 | 75 | 145 | 126 | 293 | 1247 | 19 |  |
| **Total cases** | **409** | **1359** | **641** | **1749** | **375** | **3162** | **6060** | **2769** | **10148** | **102354** | **1193** | **23801** |
| **Yearly deaths** | 0 | 6 | 1 | 2 | 0 | 0 | 14 | 8 | 26 | 179 | 3 | 98 |

* Data available up to 16 September 2021
