# Supplementary material for: COVID-19 onslaught is masking the 2021 dengue outbreak in Dhaka, Bangladesh
Source: PLoS Negl Trop Dis. 2022 Jan 20;16(1):e0010130. doi: 10.1371/journal.pntd.0010130 (PMC8775334; doi:10.1371/journal.pntd.0010130)
Supplement: S1 Fig — (DOCX) [file pntd.0010130.s002.docx]

**S1 Fig:** Cumulative trend of dengue cases over a period of 2010-2020
